# Circulating Biomarkers for the Prediction of Abdominal Aortic Aneurysm Growth

**DOI:** 10.3390/jcm10081718

**Published:** 2021-04-16

**Authors:** Petroula Nana, Konstantinos Dakis, Alexandros Brodis, Konstantinos Spanos, George Kouvelos

**Affiliations:** 1 Vascular Surgery Department, Larissa University Hospital, Faculty of Medicine, School of Health Sciences, University of Thessaly, Mezourlo, 41110 Larissa, Greece; kostasdakis1994@gmail.com (K.D.); spanos.kon@gmail.com (K.S.); geokouv@gmail.com (G.K.); 2 Department of Neurosurgery, Larissa University Hospital, Faculty of Medicine, School of Health Sciences, University of Thessaly, Mezourlo, 41110 Larissa, Greece; alexgbrodis@yahoo.com

**Keywords:** abdominal aortic aneurysm, biomarkers, aneurysm growth

## Abstract

Background: Abdominal aortic aneurysm represents a distinct group of vascular lesions, in terms of surveillance and treatment. Screening and follow-up of patients via duplex ultrasound has been well established and proposed by current guidelines. However, serum circulating biomarkers could earn a position in individualized patient surveillance, especially in cases of aggressive AAA growth rates. A systematic review was conducted to assess the correlation of AAA expansion rates with serum circulating biomarkers. Methods: A data search of English medical literature was conducted, using PubMed, EMBASE, and CENTRAL, until 7 March 2021, in accordance with the Preferred Reporting Items for Systematic Reviews and Meta-Analysis statement (PRISMA) guidelines. Studies reporting on humans, on abdominal aortic aneurysm growth rates and on serum circulating biomarkers were included. No statistical analysis was conducted. Results: A total of 25 studies with 4753 patients were included. Studies were divided in two broad categories: Those reporting on clinically applicable (8 studies) and those reporting on experimental (17 studies) biomarkers. Twenty-three out of 25 studies used duplex ultrasound (DUS) for following patients. Amongst clinically applicable biomarkers, D-dimers, LDL-C, HDL-C, TC, ApoB, and HbA1c were found to bear the most significant association with AAA growth rates. In terms of the experimental biomarkers, PIIINP, osteopontin, tPA, osteopontin, haptoglobin polymorphisms, insulin-like growth factor I, thioredoxin, neutrophil extracellular traps (NETs), and genetic factors, as polymorphisms and microRNAs were positively correlated with increased AAA expansion rates. Conclusion: In the presence of future robust data, specific serum biomarkers could potentially form the basis of an individualized surveillance strategy of patients presenting with increased AAA growth rates.

## 1. Introduction

Despite abdominal aortic aneurysm (AAA) being an asymptomatic entity, rupture complicates this silent pathology with a high mortality risk. Aneurysm identification on incidental imaging or screening programs at an early stage and small diameter allows for a close surveillance and repair [1]. However, not all aneurysms expand with the same rate and are not associated with the same risk of rupture, while diameter cannot always predict the physical evolution of an AAA [2,3,4]. A plethora of studies using imaging modalities and AAA anatomical characteristics tended to define models that could describe the expansion model of small or larger AAAs [5,6,7,8]. From ultrasonography to modern mathematical flow models, different methods have been used to identify these markers that could eliminate this group of patients needing closer re-evaluation and earlier management [9].

As different anatomical characteristics recorded on imaging modalities have been associated with aneurysm expansion, an analogous interest exists regarding the application of biomarkers that could identify AAA growth [10,11]. However, important discrepancies exist among the available studies [11]. A large spectrum of biomarkers is recorded in the current literature, from the commonly applied clinical circulating biomarkers to more specific sophisticated genetic models that could be used to evaluate AAA expansion rate [12]. The need to predict aneurysm evolution and if possible, to hamper sac expansion, is of high interest, as this approach would permit a closer surveillance screening and a more individualized therapeutic approach.

Along this line, a systematic review was conducted to present the existing evidence of different circulating biomarkers that may have a potential role on AAA growth prediction.

## 2. Materials and Methods

### 2.1. Eligible Studies

**Figure 1:**
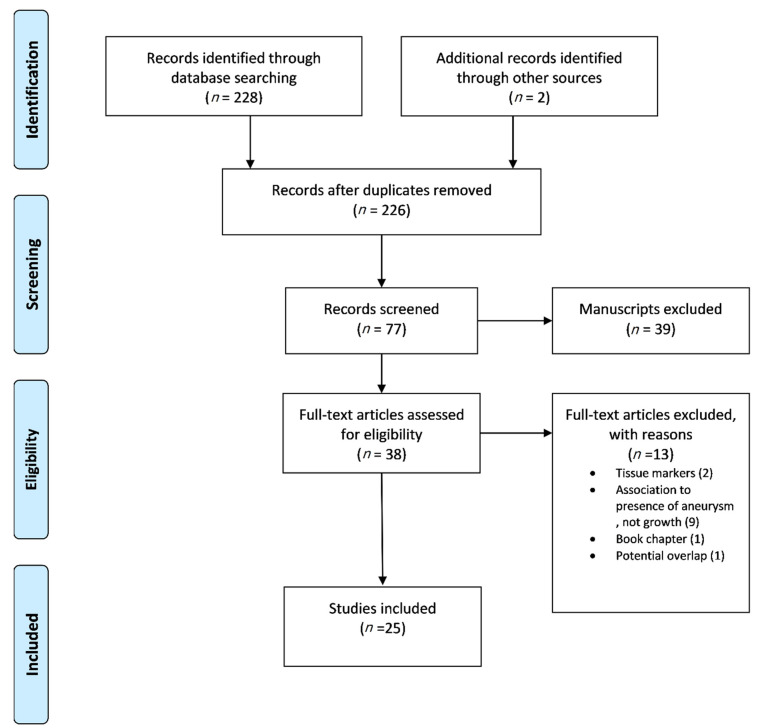
The flow chart of the selection process according to PRISMA statement.

The Preferred Reporting Items for Systematic Reviews and Meta-analyses (PRISMA) guidelines were followed [13]. Studies of English medical literature, reporting data on the evaluation of biomarkers (see Section 2.5), regarding their potential role on the identification of AAA growth (see Section 2.5) on humans, were considered eligible. Studies referring to data based on animal studies, any other aortic pathology besides AAA, and non-plasma circulating biomarkers (unavailable by venipuncture) were excluded. Scientific council approval in terms of ethical considerations was not required due to the nature of the study. Data extraction and methodological assessment was carried out by two independent investigators (P.N., K.D.). Any discrepancy was resolved after consultation by a senior investigator (G.K.). Consequently, a full-text review of the eligible studies was conducted, respecting the eligibility and exclusion criteria (Figure 1).

### 2.2. Search Strategy

A data search of English medical literature was conducted, the endpoint being 7 March 2021. The established medical databases PubMed, EMBASE, and CENTRAL were searched under the patient/population, intervention, comparison and outcomes (PICO) model, in order to determine the clinical questions and select the appropriate articles (Supplementary Table S1) [14]. The following search terms including Expanded Medical Subject Headings (MeSH) were used in various combinations: Abdominal aortic aneurysm, growth, biomarker. Primary selection was constructed on titles and abstracts, while a secondary investigation was executed based on full texts.

### 2.3. Data Extraction

A standard Microsoft Excel extraction file was developed. Extracted data included general data such as article author, year of publication, study period, journal of publication, and type of study. In addition, clinical data extracted from text or tables included the number of patients included, cohort characteristics, biomarker in evaluation, method of biomarker assessment, growth rate definition in each study, type of imaging used, correlation of biomarker to AAA growth, and statistical significance.

### 2.4. Quality Assessment

Quality assessment for individual studies and risk of bias evaluation was addressed using the ROBINS-I tool [15] for observational, non-randomized studies and the RoB-II tool [16] for randomized, controlled studies. Observational studies were judged as bearing a “Low”, “Moderate”, “Serious”, or “Critical” risk of bias, based on 7 domains, while RCTs were evaluated bearing a “Low”, “Some concerns”, or a “High” risk of bias, based on 5 domains (Supplementary Table S2). Risk of bias evaluation was carried out by two independent investigators (P.N., K.D.). In cases of disagreement, a third author was advised (G.K.).

### 2.5. Definitions

A biomarker was considered a characteristic that is objectively measured and evaluated as an indicator of normal biological processes, pathogenic processes, or pharmacologic responses to a therapeutic intervention, as defined by the biomarkers.

### 2.6. Definitions Working Group 

AAA growth was considered as the difference among two measurements of the maximal anteroposterior diameter of a diagnosed abdominal aortic aneurysm, based on measurements achieved either by ultrasonography (US), computed tomography (CT) or magnetic resonance imaging (MRI), between two set timepoints, at least 12 months apart or more. AAA growth was measured as mm/year [17].

### 2.7. Statistical Analysis

Only descriptive data were presented, because this systematic review did not aim to compare the efficacy of biomarkers on AAA growth.

## 3. Results

Twenty-five studies with 4753 patients were included in this systematic review. To facilitate data presentation, the studies were divided into two groups. The first group included studies assessing clinically applicable biomarkers and the second group included studies recording data on experimental biomarkers not used in the daily clinical practice.

Eight studies presenting data on clinical biomarkers were included; one randomized control trial [18], 3 prospective [19,20,21], and 4 retrospective [22,23,24,25] observational studies, published between 2008 and 2018 (Table 1). All analyses assessed patients that underwent screening controls or were hospital referrals and presented an AAA of more than 30 mm of diameter (range 30–50 mm). Considering experimental circulating biomarkers, 17 articles were included, all presenting results from prospective [26,27,28,29,30,31,32,33,34,35,36,37,38,39,40,41] observational studies, except one retrospective [42] analysis (Table 2). In total, 3152 patients with AAA of more than 30 mm were included (range 30–49 mm).

Among studies presenting clinical biomarkers, the most commonly applied one was D-dimers which was assessed in three studies [20,21,25]. D-dimers’ role as an indicator of the process of thrombosis and thrombolysis and their known association with other cardiovascular entities has been assessed to further identify their potential impact on AAA evolution. The lipidemic biomarkers (total cholesterol [18], apolipoprotein-B [18], low density lipids (LDL) [18] and high-density lipids (HDL) [22]) and C-reactive protein (CRP) [19] have been used due to their proven role on the process of atherothrombosis and their relationship to a higher-risk of cardiovascular events. In one study, the role of HbA1 c was addressed due to the already known negative association between diabetes and AAA pathogenesis [24]. All the markers assessed in the studies, as well as the potential underlying etiopathologic relationship between them and AAA evolution is presented in Table 3. In the experimental group, a variety of biomarkers was applied to identify prediction models in AAA evolution (Table 4). Nine out of them participate in the inflammation cascade while 3 are associated with the physiological coagulation mechanisms and 2 on the degenerative procedures of the tissues. Neutrophil gelatinase-associated lipocalin was studied as an indicator of the inflammatory degenerative process of aneurysm formation [34,42] and evolution while the potential role of insulin-like growth factor I (IGF-I) and II was analyzed under the spectrum of the negative association between diabetes and AAA [33]. The etiopathological association of all experimental markers and AAA growth is also presented in Table 4.

For the evaluation of aneurysm growth, duplex ultrasonography (DUS) was used in the vast majority of the studies (23 out of 25 studies) [18,19,20,21,22,23,24,25,26,27,28,30,31,32,33,34,35,36,37,38,39,40,42]. Aneurysm growth definition varied among studies. The change in the antero-posterior diameter of the aneurysm sac during the whole observation period divided by the aforementioned time interval (diameter at the latest evaluation-diameter at the initial evaluation/time interval in years) was used in 15 studies to identify the annual growth rate of the aneurysm. All approaches used to evaluate AAA growth rate are presented in Table 3 and Table 4.

D-dimers were positively associated in 3 studies with aneurysm growth with an associated statistical significance. In accordance, the lipidemic markers played a predictive role in AAA expansion; total cholesterol and apolipoprotein-B had a positive relation while HDL presented a negative association; higher HDL levels were associated with lower AAA growth. The negative association of diabetes and AAA pathogenesis was detected by the negative correlation between HbA1 and expansion rate. The association between clinical biomarkers and AAA growth is presented in Table 5. Among the experimental biomarkers, amino-terminal propeptide of type III procollagen (PIIINP) [26], tissue-type plasminogen activator (tPA) [27,29,40], osteopontin [34], haptoglobin polymorphism [30], IGF I and II [33], thioredoxin (TRX) [31], neutrophil extracellular traps (NETs) [41], and genetic factors, as polymorphisms [35] and micro RNAs [36] were positively associated with aneurysm expansion. Two studies reported no association between NGAL and AAA growth [34,42]. All data regarding the experimental markers are available in Table 6.

### Risk of Bias Evaluation

Twenty-four observational studies were assessed on 7 domains (ROBINS-I tool), while 1 RCT was assessed on 5 domains (RoB-II tool). Twenty-one [19,20,21,22,23,25,27,28,29,30,31,32,33,34,35,36,37,38,39,40,41] out of 24 observational studies were attributed a “Moderate” risk of bias, while the rest 3 [24,26,42] were attributed a “Serious” risk of bias (Supplementary Table S2). The RCT [18] was attributed a “Some Concerns” risk of bias grade. Confounders on which the studies were judged included consistency and control of method of biomarker evaluation, potential subgroup analysis of patients, method of imaging technique, and number and experience of imaging techniques operators.

## 4. Discussion

AAA represent a category of vascular lesions with high morbidity and mortality, especially in the case of aneurysm rupture. Current guidelines suggest elective repair based mainly on aneurysmal diameter and/or other characteristics of the AAA [8,43]. Proposed screening strategies vastly stand on imaging techniques, including mainly DUS, adhering to the phenomenon of increased rupture risk in patients of specific demographic attributes and AAA diameter [44]. Studies have shown that patients with particular aneurysmal attributes would be acceptable surgical candidates, especially for endovascular interventions, even if AAA diameter has not achieved the diameter’s threshold [45,46]. While AAA growth is observed through typical, time-set imaging follow-up, stratification of high-risk patients with expeditious AAA growth, through serum biomarkers, could be a valid approach for individualized imaging surveillance. These patients could benefit from a rather targeted surveillance approach as well as an early endovascular or open surgical repair.

The pathogenesis of AAAs advocates for an extensive list of serum circulating or histologically detected biomarker candidates. Each category bears an important role in the different phases of the natural history of AAA [47,48,49]. Biomarkers detected through histological evaluation of an AAA open surgical repair specimen do not conform with the concept of preoperative surveillance and disease progression and therefore cannot be used in clinical practice. However, serum circulating biomarkers appertaining to recognized pathophysiologic processes of AAA pathogenesis, including thrombosis, inflammation, extracellular matrix (ECM) degradation, lipid metabolism, as well as genetic predisposition, could potentially form the basis of a stratification screening or surveillance strategy for patients in need of more frequent follow-up.

As proposed by many studies, certain mediators or by-products of thrombosis and lipid metabolism have been linked to AAA growth. These biomarkers can be easily and cost-effectively implemented in everyday clinical practice [18,19,20,22,25]. D-dimers, a known fibrin degradation by-product, has been shown to be associated with AAA expansion, as higher levels have been correlated with increased growth rate. Correlation of other thrombosis-related biomarkers, including PAP complex [21,50], homocysteine [51], and TAT [25], has also been reported. Higher levels of HDL-C, a biomarker related to lipid metabolism, have been correlated with decreased AAA growth rates in a screening population [22]. Furthermore, increased levels of total cholesterol and apolipoprotein B, both markers easily quantified and major constituents of lipid metabolism, have been associated with increased growth rates of AAA [18]. On the other hand, given the potentially protective nature of diabetes mellitus in AAA, glycated hemoglobin (HbA1c) has been studied as a possible biomarker of inverse association with AAA expansion [52,53,54,55]. A lower growth rate was observed in patients with higher HbA1c levels; 1.8 mm/year decrease of rate in HbA1c 44–77 compared to 28–39 mmol/mol [24]. The recognized correlations of the abovementioned biomarkers, in addition to their cost-effectiveness and their wide-spread use in everyday clinical practice, renders them attractive candidates for future studies aiming to provide robust data on their relation to AAA expansion rates.

Concurrently, a plethora of less utilized biomarkers correlating to various stages of AAA progression have been studied, posturing as alluring secondary candidates. Firstly, extracellular matrix components and degradation enzymes have been associated with AAA growth rate. The well-defined role of elastin, biglycan, and type III collagen in the structural integrity of the aortic wall provided the basis for studies reporting data on the by-products of these proteins associated with ΑΑΑ progress and increased sac expansion [18,26,29,56,57]. Inadvertently, extracellular matrix proteinases (MMP-2, MMP-9 [58], cathepsins B, D, L, and S [59]) responsible for ECM cleavage, and proteinases inhibitors (a1-antithrypsin [19], cystatin-B [37], cystatin-C [60]) play a significant role in the aortic wall remodeling occurring in AAA pathogenesis with several studies revealing either positive or inverse correlations with AAA growth rates. An abundance of modulators and mediators expressing the inflammatory and oxidative processes have also been studied with conflicting outcomes [31,32,38,61,62]. Synchronously, studies on promising novel biomarkers requiring genome sequencing analysis have been conducted, with propitious results. Specifically, genomic DNA analysis of genetic polymorphisms showed increased risk of aggressive-growth over slow-growth AAA [36,41,63,64,65]. Current data on these aforementioned biomarkers are promising, despite the fact that firm conclusions cannot be provided. Interestingly, calprotectin, a protein commonly associated with inflammatory cells (neutrophil granulocytes, monocytes, macrophages), has been related to AAA pathogenesis. These results provide further solid ground for future trials, aiming to assess the relation between the antimicrobial protein and AAA growth rate [66,67]. As the knowledge on AAA pathogenesis increases, novel studies may offer validated markers that could be used for the detection of this high-risk group of patients while pharmaceutical factors may provide a conservative management on AAA presence and expansion.

### Limitations

The strength of the current review is limited by a series of factors. Firstly, the retrospective nature of the included studies confines its ability to reach pertinent results. Secondly, vast incoherencies among studies in terms of the types of biomarker assessed, studied population and cohorts, lack of control groups, follow-up intervals, and standardized methodological evaluations (imaging techniques, biomarkers quantification methods) impede the production of robust results, as well as the ability of quantitative analysis of the said results. Finally, most studies were judged as having “Moderate” risk of bias, mainly due to selection bias and inadequate confounder control.

## 5. Conclusions

Blood circulating biomarkers may offer a valid approach in the future for the detection of AAA expansion. The current literature provides a plethora of data with conflicting results and firm conclusions cannot be provided. In the presence of future robust data, specific serum biomarkers could potentially form the basis of an individualized surveillance strategy of patients presenting with increased AAA growth rates.

## Figures and Tables

**Table 1:** General characteristics of studies on circulating clinical biomarkers.

Author	Year of Publication	Journal	Type of Study	Number of Patients	Study Cohort
Ceniga et al. [19]	2009	European Journal of Vascular and Endovascular Surgery	Prospective observational (case-control)	70	35 patients (AAA 30–49 mm)—35 healthy controls (AAA < 30 mm)
Colledge et al. [20]	2011	European Heart Journal	Prospective observational (case-control)	299	299 patients (AAA 30–40 mm) (261; AAA 30–39 mm—38; AAA 40–49 mm)
Ceniga et al. [21]	2014	Annals of Vascular Surgery	Prospective observational (case ceries)	96	96 patients (AAA 30–49 mm)
Burillo et al. [22]	2015	Thrombosis and Hemostasis	Retrospective observational (case-control)	122	122 patients (AAA > 30 mm) (86; AAA 30–49 mm—36; AAA > 50 mm)
Moxon et al. [23]	2016	Atherosclerosis	Retrospective observational (case-control)	250	250 Patients (AAA 30–39 mm) (65; AAA with iron overload (Ferritin > 200 ng/mL)—185 without IO)
Kristensen et al. [24]	2017	Arteriosclerosis, Thrombosis, and Vascular Biology	Retrospective observational (case-ontrol)	319	319 patients (AAA 30–49 mm) (61 AAA with DM—258 without DM)
Deeg et al. [18]	2016	Current Medical Research and Opinion	Randomized controlled trial (RCT)	93	93 patients (AAA 35–50 mm) (44 AAA with doxycycline—49 AAA without doxycycline)
Sundermann et al. [25]	2018	Blood Advances	Retrospective observational (case-control)	352	169 patients (AAA > 25 mmm) (84 slow growing AAA, <2 mm/year, 85 fast growing AAA, >2 mm/year)—68 subaneurysmal aorta (25–29 mm)—115 healthy control (AA < 25 mm)

All analyses assessed patients that underwent screening controls or were hospital referrals and presented an AAA of more than 30 mm of diameter. Notes: AAA: abdominal aortic aneurysm; DM: diabetes mellitus.

**Table 2:** General characteristics of studies on circulating experimental biomarkers.

Author	Year of Publication	Journal	Type of Study	Number of Patients	Study Cohort
Satta et al. [26]	1997	Journal of Vascular Surgery	Prospective observational (case-control)	139	139 patients with AAA under surveillance and or hospital referrals (AAA > 40 mm)
Lindholt et al. [27]	2003	European Journal of Vascular & Endovascular Surgery	Prospective observational (case series)	70	70 patients from the Viborg cohort (screening program) (AAA 30–49 mm)
Colledge et al. [28]	2007	Arteriosclerosis, Thrombosis and Vascular Biology	Prospective observational (case-control)	198	146 patients from the Western Australia Screening Study and 52 referrals from a tertiary hospital (AAA > 30 mm)
Flondell–Site et al. [29]	2010	Vascular and Endovascular Surgery	Prospective observational (case series)	397	178 patients with AAA, referrals at the Malmo Hospital and 219 control healthy individuals
Wiernicki et al. [30]	2010	Journal of Vascular Surgery	Prospective observational (case series)	83	83 patients with AAA under surveillance (AAA diameter, non-applicable)
Martinez–Pinna et al. [31]	2010	Atherosclerois (TRX)	Prospective observational (case series)	166	88 patients from a Spanish screening cohort and 78 patients from the Viborg cohort (screening program) (AAA > 30 mm)
Martin–Ventura et al. [32]	2011	Atherosclerois (sTWEAK)	Prospective observational (case series)	150	43 patients with AAA vs. 28 healthy controls and 79 patients from the Viborg cohort (AAA 30–49 mm)
Lindholt et al. [33]	2011	European Journal of Vascular & Endovascular Surgery (IGF)	Prospective observational (case series)	115	115 patients with AAA from a screening program (AAA > 30 mm)
Ramos–Mozo et al. [34]	2012	Atherosclerois (NGAL)	Prospective observational (case series)	100	100 patients with AAA under surveillance from a screening program (AAA median diameter 37.5 mm)
Ye et al. [35]	2016	Atherosclerois(SNPs)	Prospective observational (case series)	651	651 patients participants in the Mayo Clinic Vascular Disease Biorepository, with AAA with at 2 least to diameter measurements on surveillance program (AAA > 30 mm or with a history of open or endovascular AAA repair)
Wainhanen et al. [36]	2017	Atherosclerois	Prospective observational (case-control)	242	192 patients with AAA and 50 healthy controls from a screening program and hospital referral (AAA > 30 mm)
Wang et al. [37]	2018	European Journal of Vascular & Endovascular Surgery	Prospective observational (case-control)	749	551 male patients with AAA and 198 age-matched healthy controls from the Viborg cohort (screening program) (AAA > 30 mm)
Ahmad et al. [38]	2018	European Journal of Vascular & Endovascular Surgery	Prospective observational (case series)	97	97 patients—NHS referrals under surveillance for AAA (mean diameter 39 mm)
Groeneveld et al. [42]	2019	Annals of Vascular Surgery	Retrospective observational (case series)	7	7 patients from hospital referrals with intact AAA (AAA diameter: Non-applicable)
Lindholt et al. [39]	2020	Journal of Vascular Surgery	Prospective observational (case-control)	692	504 male patients with AAA and 188 healthy controls in the Viborg cohort (screening program) (AAA > 30 mm)
Memon et al. [40]	2020	European Journal of Preventive Cardiology	Prospective observational (case-control)	170	134 patients with AAA and 136 healthy control from a screening program (AAA > 30 mm)
Eilenberg et al. [41]	2021	Translational Research	Prospective observational (case series)	28	28 patients with AAA patients under surveillance from the Vienna General Hospital (AAA diameter: Non-applicable)

**Table 3:** Specific characteristics of studies on circulating clinical biomarkers.

Author	Aim	Etiology	Biomarker	Method	Growth Rate Definition	Type of Imaging
Ceniga et al. [19]	Identification of possible association of CRP, Α1at, Lpa with AAA growth	CRP; independet risk factor for atherosclerosis, CVD, symptomatic/ruptured AAA, Α1at; conflicting data on CVD, Lpa; established role in CVD, CHD, atherothrombosis, stroke	CRP, A1at, Lpa	CRP, Lpa; immunoturbidimetric method, A1at; immunonephelometric method	Millimeter difference of maximum transverse and anteroposterior external diameters of the infrarenal aorta, perpendicular to the aortic axis, in ultrasound/CT scans taken 12 months apart (mm/year); (FUP: 1 year)	DUS (AAA 30–39 mm), CT (AAA 40–49 mm)
Colledge et al. [20]	Identification of possible association of D-dimers, CRP, creatinine in AAA growth	D-dimers; indicator of thrombosis-thrombolysis (DVT, CVD, AAA)	D-dimers	D-dimers: ELISA or latex-enhanced immunoassay	Millimeter difference of the greatest diameter of the infrarenal aorta in ultrasound scans every 6 or 12 months (mm/year). (Median FUP: 5.5 years)	DUS
Ceniga et al. [21]	Identification of possible association of various biomarkers in AAA growth rate	D-dimers; indicator of thrombosis-thrombolysis (DVT, CVD, AAA), cystatin-C; recent biomarker of CVD (MCI, stroke, HF, PAD), PAP complex; possible role in atherosclerosis	D-dimers, cystatin-C, A1at, MMP2, MMP9, myeloperoxidase, MCP-1, homocysteine, PAP complex, CRP	D-dimers; ELISA, cystatin-C; N latex cystatin C assay, PAP complex; ELISA	Millimeter difference of measured maximum outer-to-outer transverse, anteroposterior, and lateral aortic diameters, perpendicular to the aortic axis, in US/CT scans taken 12 months apart (mm/year); (FUP: 1 year)	DUS (AAA 30–39 mm), CT (AAA 40–49 mm)
Burillo et al. [22]	Identification of possible association of HDL-C to AAA growth	HDL-C; inversily associated with CVD, atheromatosis, atherothrombosis	HDL-C	ELISA	Millimeter difference of measured maximal perpendicular anteroposterior diameter, in US/CT scans taken 12 months apart (mm/year); mean FUP: 8.1 years	DUS (AAA 30–50 mm), CT (AAA > 50 mm)
Moxon et al. [23]	Identification of possible assocation between ferritin and AAA diagnosis, size, growth	Ferritin; marker of CVD, iron deposition in histologic examination of AAA	Ferritin	ELISA	Yearly AAA growth rate was calculated as a percentage of the size of the AAA in the preceding year (e.g., AAA growth rate between screening and year 1 rescan = 100 × (year 1 AAA diameter/AAA diameter at screening)-1)	DUS
Kristensen, et al. [24]	Identification of possible association of HbA1c to AAA growth	HbA1c; risk factor for atherosclerosis, CVD—possible protective factor in AAA pathogenesis	HbA1c	Not stated	Millimeter difference in maximal anterior-posterior AAA diameter in US scans taken 12 months apart (mm/year); mean FUP: 3.88 years	DUS
Deeg et al. [18]	Identification of possible association of various biomarkers in AAA growth rate between AAA patients receiving and not receiveing doxycycline	TC, LDL, biglycan; atherosclerosis and CVD association, elastin products; degradation products associated with AAA pathogenesis	Total cholesterol, ApoB, elastin DP, biglycan DP, cathrepsin S, LDL	Multi-analyte profiling technology	Millimeter difference of measured maximal anteroposterior AAA diameter, perpendicular to the blood flow, from inner-to-inner wall, in US scans taken 6–12–18 months apart (mm/month); FUP: 18 months	DUS
Sundermann et al. [25]	Identification of possible association of D-dimers, TAT, PF4 with AAA stratification and growth	D-dimers; indicator of thrombosis-thrombolysis (DVT, CVD, AAA), TAT; increased levels in CD, stroke, PAD, atherothrombosis, PF4; inflammatory/coagulative role in AAA thrombus	D-dimers, TAT complex, PF4	ELISA	Millimeter difference of measured maximum AAA diameter, in US scans taken 12 months apart (mm/year); slow-growing: <2 mm/year; fast-frowing: > 2 mm/year	DUS

All the markers assessed in the studies, as well as the potential underlying etiopathologic relationship between them and AAA evolution, are presented. Notes: AAA: Abdominal aortic aneurysm; CD: Coronary disease; CHD: Cardiac heart failure; CT: Computed tomography; CVD: Cardiovascular disease; DVT: Deep vein thrombosis; HF: Heart failure; MI: Myocardial infarction; FUP: Follow-up; PAD: Peripheral arterial disease; US: Ultrasonography.

**Table 4:** Specific characteristics of studies on circulating experimental biomarkers.

Author	Aim	Etiology	Biomarker	Method	Growth Rate Definition	Imaging
Satta et al. [26]	Identification of the association between serial changes in the concentration of the aminoterminal propeptide of type III procollagen (PIIINP) in serum to the rate of AAA growth	PIIINP; synthesis of type III collagen; increased turnover of in patients with AAAs when compared with patients with atherosclerotic aorto-iliac occlusive disease	Serum PIIINP	Equilibrium-type radioimmunoassays	No definition recorded in text (FUP:NA)	DUS
Lindholt et al. [27]	Identification of the activating pathways of plasminogen as predictors of AAA progression	tPA, IgA and CP and S-cotinine; markers of fibrinolytic function in plasma and activation of the degenerative processes in tissues-proteolysis; plasmin and antiplasmin complex (PAP) correlates with aneurysmal expansion and prediction for cases expanding to operation recommendable sizes	tPA, IgA ± CP, and S-cotinine	ELISA	Change in anterior-posterior diameter during the whole observation divided by years in annual units (FUP: 3.5 years)	DUS
Colledge et al. [28]	Identification of the relationship between serum osteopontin (OPN) concentrations, polymorphisms of the OPN gene, and AAA presence and growth in humans	OPN: Bone remodeling, vascular calcification, and tumor metastasis; inflammation, proteolysis, and atherosclerosis, integral processes in AAA and animal models	OPN (osteopontin)	ELISA	Maximum transverse and anteroposterior diameter measurements (FUP: 3 years)	DUS
Flondell–Site et al. [29]	Quantification plasma metalloproteinases (MMP)-2 and -9 and their endo-genous tissue inhibitors (TIMP-1, serpine-1, tPa-serpine-1, and the APC-PCI complex) in patients with AAA and their relationship between these markers and AAA diameter and growth	APC-PCI; physiological inhibitors of free tissue plasminogen activator (tPA) with proteolytic and fibrinolytic activity; increased concentrations of the APC-PCI complex in patients with AAA and imbalances between MMPs and their inhibitors have been reported to occur in AAA	APC-PCI (acivated C protein–protein C inhibitor), MMP-2, MMP-9, and their endo- genous tissue inhibitors, TIMP-1, serpine-1, tPa-serpine-1	Biotrak activity assay systems, ELISA, DELFIA	Change in anterior-posterior diameter during the whole observation divided by years in annual units (FUP: 7 years)	NA
Wiernicki et al. [30]	Identification of the association between haptoglobin polymorphism (Hp) phenotype to AAA growth rate and assessment of serum elastase activity and markers of inflammation in patients with newly diag- nosed AAA	Hp gene; hemoglobin-binding protein expressed by a genetic polymorphism as three major phenotypes: Hp 1-1, Hp 2-1, and Hp 2-2; suppression of inflammatory responses; influences the dilatation of the abdominal aorta and probably has a direct effect on the degradation of elastin in the atherosclerotic aorta	HP (haptoglobin polymorphism)	Starch-gel electrophoresis	Growth rate identified as mm/y = max diameter at last scan- max diameter at first scan/time interval (FUP:NA)	DUS
Martinez–Pinna et al. [31]	Identification of thiorexodin (TRX) in conditioned media from the different layers of AAA thrombus, and in serum of patients with AAA	TRX; reactive oxygen species in inflammation and hemolysis in luminal layer of the thrombus; intracellular antioxidant enzyme elevated in CAD and intraplaque hemorrhage	Thioredoxin (TRX)	ELISA	Change in anterior-posterior diameter during the whole observation divided by years in annual units (FUP: NA)	DUS
Martin–Ventura et al. [32]	Identification of the association of the soluble tumor necrosis factor-like weak inducer of apoptosis (sTWEAK) with AAA growth rate	sTWEAK; cellular growth, proliferation, migration, osteoclastogenesis, angiogenesis, apoptosis; marker of CVD, CAD, carotid stenosis, and PAD that may have an association to AAA	sTWEAK (soluble tumor necrosis factor-like weak inducer of apoptosis)	ELISA	Change in anterior-posterior diameter during the whole observation divided by years in annual units (FUP: 5 years)	DUS
Lindholt et al. [33]	Estimation of the potential role of insulin-like growth factor I (IGF-1) and IGF-2 as biomarkers for AAA	IGF-1; mediator of CVD; vascular protective factor in AAA	Insulin-like growth factor 1 (IGF-1) and 2 (IGF-2)	Validated, in-house time-resolved immunofluorometric assays	Change in anterior-posterior diameter during the whole observation divided by years in annual units (FUP: 10 years)	DUS
Ramos–Mozo et al. [34]	Identification of the relationship of NGAL concentrations in the plasma of three groups of patients and related them to the presence, size and growth of AAAs	Plasma NGAL; neutrophil-derived protein during inflammation; marker of CV risk factors in asymptomatic atherosclerosis	NGAL (neutrophil gelatinase-associated lipocalin)	ELISA	NA	DUS
Ye et al. [35]	Association of multi-locus generic risk score (GRS) based on single nucleotide polymorphisms (SNPs) associated with AAA in genome-wide association studies (GWAS) with AAA growth prediction beyond conventional risk factors	Genome-wide association studies (GWAS); several common single nucleotide polymorphisms (SNPs); known association with AAA presence	Multi-locus GRS	Illumina Infinium Human core Exome Array, and Illumina Human 610 and 660 W Quad-v1	Latest assessed diameter (mm)/pre-operation minus first diameter(mm)/time interval (years) (FUP: NA)	DUS, CT, MRI, DSA
Wainhanen et al. [36]	Associations between a wide range of micro-RNAs (miRNAs) and presence and growth of AAA	Circulating miR; 8–22 nucleotide short non-coding RNAs secreted by cells that regulate expression of target genes by interfering with transcription or inhibiting translation; associated with AAA presence (miR-155, miR-191-3p, miR-455-3p, miR-1281, and miR-411)	microRNA (miRNA)	miRCURYTM RNA isolation kit-biofluids/PCR	NA (FUP: NA)	DUS
Wang et al. [37]	Association of plasma cystatin B with AAA presence, size, growth rate, or need for later surgical repair	Cystatin B; marker of human malignant tumors in lipopolysaccharide (LPS) activated human blood monocytes and in interferon-g induced mouse macrophages, cystatin B deficiency in mice or loss of function mutation in humans associated with neurological dysfunction; aortic wall weakening process is mediated by proteases, including cysteinyl cathepsins, negative correlation of human plasma cystatin C levels with AAA size and annual expansion rate	Cystatin B	ELISA	Change in anterior-posterior diameter during the whole observation divided by years in annual units (FUP: 5 years)	DUS
Ahmad et al. [38]	Investigation of the relationship between serum interleukin (IL-1α) levels and asymptomatic infrarenal AAA growth rates, absolute size, and morphology	Il-1α; pro-inflammatory cytokine not normally detectable in the circulation in health individuals; non-specific CVD marker; marker of aortic disease related to AAA diameter	IL-1α	Boster immunoassay kit	Absolute (mm) change in maximum AP diameter and absolute (mm/year) change in maximum AP diameter over time (FUP:NA)	DUS
Groeneveld et al. [42]	Investigation of the role of neutrophil gelatinase-associated lipocalin (NGAL) in AAA development and rupture	NGAL; acute phase protein stored in neutrophils, diagnostic and prognostic tool for several CVD; potential prevention of metaloproteinase (MMP-9) from inactivation and aortic wall degeneration; NGAL inhibition in mice attenuation of AAA growth, protective role against apoptosis	NGAL (neutrophil gelatinase-associated lipocalin)	ELISA	NA (multiple measurements to assess the expansion rate, FUP: NA)	CT or DUS
Lindholt et al. [39]	Investigation of the potential role of plasma microfibrillar- associated protein 4 (pMFAP4) as a biomarker of AAA	MFAP4; factor expressed in human elastic fibers in blood vessels, induction of smooth muscle cell proliferation and migration and monocyte chemotaxis; marker of tissue remodeling-related diseases	Microfibrillar- associated protein 4 (MFAP4)	AlphaLISA	Change in anterior-posterior diameter during the whole observation divided by years in annual units (FUP: 5 years)	DUS
Memon et al. [40]	Identification of diagnostic and prognostic biomarkers for AAA diameter and growth	MPO, tissue-type plasminogen activator (t-PA), CSTB; proteolytic and fibrinolytic activity; pathophysiologic marker of CVDs	Myeloperoxidase (MPO), tissue- type plasminogen activator (t-PA), and cystatin-B (CSTB)	Proseek Multiplex CVD III96 × 96 panel	NA	DUS
Eilenberg et al. [41]	Identification of the diagnostic or prognostic role of neutrophil extracellular traps (NETs) in AAA patients	NETs; marker of excessive neutrophil activation in destruction of pathogens; marker of AAA development in a mouse model by propagating the local immune reaction in aneurysm tissue	Neutrophil extracellular traps (NETs)	ELISA	The AAA maximum diameter is measured with semi-automatic tools with mean intra- and interobserver variability ranging at 0.13 and 0.27 mm, respectively	CTA

**Table 5:** The association between clinical biomarkers and AAA growth. Notes: AAA: Abdominal aortic aneurysm.

Author	Association of Biomarker to Growth	Significance	Additional Information
Ceniga et al. [19]	Α1at; positive correlation with AAA growth	r = 0.55, *p* = 0.004	CRP; no association, Lp(a); no association
Colledge et al. [20]	D-dimers; positive correlation with AAA growth: DD > 150 ng/mL -AG: 0.7 mm/DD~150–300 ng AG: 0.8/DD~300–900 ng/mL-AG: 1.3 mm/DD > 900 ng/mL-AG:1.7 mm, *p* < 0.001	r = 0.39, *p* < 0.001	Multiple linear regression analysis revealed significant positive associations of rank-transformed D-dimer (beta = 0.29, *p*, 0.001) with AAA growth
Ceniga et al. [21]	D-dimers; 1 ng/mL increase Growth 0.0062 mm/year b = 0.0062, β = 0.38 (95% CI 0.001–1.011) Adjusted R2 = 0.2)/cystatin-C; OR = 10.04 (CI 95% 1.18–85.73), PAP; OR = 1.004 (CI 95% 0.999–1.01)	D-dimers; *p* < 0.1/Cystatin-C; *p* < 0.05, PAP; *p* < 0.2	Continuous variable: Growth rate mm/year/dichotomous variable: Stability = <2 mm/year, expansion = >2 mm/year
Burillo et al. [22]	HDL-C; higher HDL-C Levels associated with lower AAA growth rate	r = −0.18, *p* = 0.07	
Moxon et al. [23]	No association	NS	
Kristensen et al. [24]	HbA1c; negative correlation with AAA growth: 1.8 mm/year (CI, 0.99–2.65; *p* < 0.000) less in HbA1c 44–77 mmol/mol vs. 28–39 mmol/mol	r = −0.177; *p* = 0.002)	
Deeg et al. [18]	Without DXC: TC, ApoB; positive correlation with AAA growth/with DXC: EP; positive correlation with AAA growth	r1 = 0.38, unadjusted *p*1 = 0.011, r2 = 0.41, unadjusted *p*2 = 0.005, r3 = 0.33, *p*3 = 0.031	
Sundermann et al. [25]	D-dimers; b = 0.21 mm/year increase per 500 ng increase, (CI 95% 0.09–0.33)/TAT; b = 0.24 mm/year increase per 1μg/mL (95% CI 0.19–0.29)/fast/slow vs. Controls for D-dimers > 500 ng OR = 7.19 (2.9–17.83)/6.23 (2.72–14.27), fast vs. slow for TAT > 4.2 μg/mL: OR 5.37 vs. 240.02	Continuous; *p* < 0.05, fast–slow; *p* < 0.001	PF4; no association

AG: Aneurysm growth; DD: D-dimers; EP: Elastin products; TAT: Thrombin-antithrombin complex.

**Table 6:** The association between experimental biomarkers and AAA growth.

Author	Association of Biomarker to Growth	Significance	Additional Information
Satta et al. [26]	Acceleration of AAA growth increased s-PIIINP correlation in the course of AAA disease (from 0.22–0.55)	*p* = 0.002 (*p* = 0.01 during the first year)	The correlation between thrombus changes and s-PIIINP tend to be lower than between diameter and s-PIIINP, except in the first year (*p* = 0.02 at the end of follow-up)
Lindholt et al. [27]	Positive correlation between annual expansion rate and tPA, IgA ± CP, and S-cotinine	r = 0.37-*p* = 0.002, r = 0.29-*p* = 0.006 and r = 0.24- *p* = 0.038, respectively	In multiple linear regression analyses adjusting for S-Cotinine, the correlation between tPA and expansion rate remained significantly correlated
Colledge et al. [28]	Serum OPN correlated with aortic diameter change	*p* < 0.001	Adjustment for other known risk factors for aortic expansion, serum OPN predicted AAA growth (*p* < 0.001)
Flondell–Site et al. [29]	No significant correlations between levels of MMP-2 or -9, TIMP-1, serpine-1, tPa- serpine-1, or the APC-PCI complex and yearly AAA growth, TIMP-1 levels independent predictors of fatal AAA rupture	NS, only for TIMP-1; *p* = 0.036	
Wiernicki et al. [30]	Hp 2-1 patients associated with significantly higher growth rate (3.69 [2.40] mm/y) of AAA compared with patients with Hp 2-2 (1.24 [0.79], *p* < 0.00001) and Hp 1-1 (1.45 [0.68], *p <* 0.00004)	*p =* 0.00001, *p =* 0.00004	Hp 2-1 associated with higher serum elastase activity and CRP concentration, Hp 2-1 phenotype only independent predictor of a higher AAA growth rate in multivariate analysis
Martinez–Pinna et al. [31]	Spearman’s correlation coefficient between TRX and AAA-growth rate. TRX predictive of patients expanding > 2 mm/year (area under ROC curve = 0.67, 95% CI, 0.55–0.79, *p* = 0.01).	*p* = 0.03	TRX optimal cutpoint of 30 ng/mL associated with a 62% sensitivity and specificity
Martin–Ventura et al. [32]	sTWEAK predictive for >2 mm/y growth rate (area under ROC curve = 0.71; 95%CI, 0.58–0.83); increase of 100 pg/mL of sTWEAK reduced risk of annual expansion rate above 2 mm by 38% (95% CI: 0.41–0.93)	*p* = 0.003 and *p* = 0.021, respectively	Inverse correlation between sTWEAK and AAA expansion rate (r = −0.263; *p* = 0.031
Lindholt et al. [33]	Positive correlation between plasma NGAL and retrospective AAA growth (rho = 0.4, *p* = 0.01), significant after adjusting for other risk factors. NGAL plasma concentration weakly associated with averaged yearly AAA growth	*p* = 0.01 and *p* = 0.2, respectively	
Ramos–Mozo et al. [34]	Serum IGF-I correlated positively with growth rate adjustment for potential confounders	*p* = 0.004	The adjusted growth rate increased by 0.53 + -0.23 mm annually between the IGF-1 tertiles (*p* = 0.013). Serum IGF-I level predicted cases needing later surgery (AOC: 0.63; 95% CI), no association of IGF-II and AAA growth
Ye et al. [35]	GRS (dichotomized by median), baseline size, diabetes, and family history associated with aneurysm growth rate (all, *p* < 0.05). Mean aneurysm growth rate 0.50 mm/year higher in those with GRS > median (5.78) than those with GRS median (*p* = 0.01), after adjustment for baseline size (*p* < 0.001), diabetes (*p* = 0.046), and family history of aortic aneurysm (*p* = 0.02)	*p* = 0.01	
Wainhanen et al. [36]	20 miRs differentially expressed between slow- and fast-growing AAAs (AUC 0.60–0.65)	*p* < 0.005	Diabetes and current smoker, together with miR-335-5p and miR- 125a-5p, with AUC of 0.84 with a specificity of 70% and sensitivity of 80%
Wang et al. [37]	In Pearson’s correlation test, plasma cystatin B not associated with AAA growth rate	*p* = 0.1	
Ahmad et al. [38]	No statistically significant relationship was detected between IL-1a and absolute AAA increase in maximum AP diameter (rho 1⁄4 0.127, *p* 1⁄4 0.214) or absolute growth rate (mm/year) (rho 1⁄4 0.123, *p* 1⁄4 0.230)	NS	
Groeneveld et al. [42]	AAA expansion rate not correlated with NGAL blood plasma (or tissue)	*p* = 0.34	
Lindholt et al. [39]	pMFAP4 significantly inversely associated with annual aneurysmal growth rate	*p =* 0.0074	No association of level of pMFAP4 in multivariate analysis
Memon et al. [40]	MPO, tissue-type plasminogen activator (t-PA) and CSTB levels significantly associated with AAA growth	*p* = 0.013, 0.016, and 0.007, respectively	MPO best prognostic value in terms of AUC (AUC, 0.71; 95% CI 0.61–0.81, with a sensitivity of 80% and specificity of 59%, higher levels of MPO (≥median) were associated with significantly faster growth of AAA median (IQR); 2.3 (2.9) mm/year) compared with lower MPO levels (median (IQR); 1.2 (1.1) mm/year)
Eilenberg et al. [41]	Prognostic value of citH3 ranged at AUROC = 0.707 (*p* = 0.015) citH3 superior to D-dimers (AUROC = 0.613, *p* = 0.186)	*p* = 0.015	194 ng/mL cut-off level for plasma citH3 to predict rapid progression (>2 mm/6 months) with 77% sensitivity und 64% specificity

AAA: Abdominal aortic aneurysm.

## Data Availability

Data sharing not applicable. No new data were created or analyzed in this study.

## Supplementary Materials

The following are available online at https://www.mdpi.com/article/10.3390/jcm10081718/s1, Table S1: PICO model, Table S2: Risk of Bias Assessment (ROBINS-I) for Observational Studies.
