# Supplementary material for: Circulating Biomarkers for the Prediction of Abdominal Aortic Aneurysm Growth
Source: J Clin Med. 2021 Apr 16;10(8):1718. doi: 10.3390/jcm10081718 (PMC8072679; doi:10.3390/jcm10081718)
Supplement: Supplementary file 1 [file jcm-10-01718-s001.zip › jcm-1185207-supplementary.pdf]

Supplementary Tables

Table S1. Patient-intervention-comparison-outcome (PICO) model.

P	Patient, population or problem	Patients with AAA under surveillance for alterations in AAA diameter
I	Intervention, prognostic factor or exposure	Serum biomarkers associated to AAA expansion rate
C	Comparison of intervention	Positive predictive role of serum biomarkers in AAA growth rate
O	Outcome you would like to measure or achieve	Changes in AAA diameter during a surveillance timespan associated to alterations on biomarkers' levels
	What type of question are you asking?	Are there clinically used serum biomarker that could predict aneurysm expansion rate in patients with AAA?
		Are there clinically used serum biomarker that could predict aneurysm expansion rate in patients with AAA?
	Type of study you want to find	Observational prospective and retrospective cohort studies, randomised control trials, Case series, case−control studies reporting on the association of serum biomarkers in AAA growth rate in humans

AAA: abdominal aortic aneurysm.

Table S2. Risk of Bias Assessment (ROBINS-I) for Observational Studies.

Studies	D1	D2	D3	D4	D5	D6	D7	Overall
Ceniga, et al [19]	(+)	(−)	(−)	(−)	(−)	(+)	(−)	(+)
Colledge, et al [20]	(+)	(−)	(−)	(−)	(−)	(+)	(−)	(+)
Ceniga, et al [21]	(+)	(−)	(−)	(−)	(−)	(+)	(−)	(+)
Burillo, et al [22]	(+)	(−)	(−)	(−)	(−)	(+)	(−)	(+)
Moxon, et al [23]	(+)	(−)	(−)	(−)	(−)	(+)	(−)	(+)
Kristensen, et al [24]	(+)	(−)	(−)	(−)	(−)	(x)	(−)	(x)
Sundermann, et al [25]	(+)	(−)	(−)	(−)	(−)	(+)	(−)	(+)
Satta, et al. [26]	(x)	(+)	(−)	(−)	(−)	(+)	(−)	(x)
Lindholt, et al. [27]	(+)	(−)	(−)	(+)	(−)	(+)	(−)	(+)

Colledge, et al. [28]	(+)	(−)	(−)	(−)	(−)	(+)	(−)	(+)
Flondell-Site, et al. [29]	(+)	(−)	(+)	(−)	(−)	(+)	(−)	(+)
Wiernicki, et al. [30]	(+)	(−)	(+)	(−)	(−)	(+)	(−)	(+)
Martinez-Pinna, et al. [31]	(+)	(−)	(−)	(+)	(−)	(−)	(−)	(+)
Martin-Ventura, et al. [32]	(+)	(−)	(+)	(−)	(−)	(−)	(−)	(+)
Lindholt, et al. [33]	(+)	(−)	(−)	(−)	(−)	(−)	(−)	(+)
Ramos-Mozo, et al. [34]	(+)	(+)	(+)	(−)	(−)	(−)	(−)	(+)
Ye, et al. [35]	(+)	(−)	(−)	(−)	(−)	(−)	(−)	(+)
Wainhanen, et al. [36]	(+)	(−)	(+)	(−)	(−)	(−)	(−)	(+)
Wang, et al. [37]	(+)	(−)	(−)	(−)	(−)	(+)	(−)	(+)
Ahmad, et al. [38]	(+)	(−)	(−)	(−)	(−)	(+)	(−)	(+)
Groeneveld, et al. [42]	(x)	(+)	(−)	(−)	(−)	(+)	(−)	(x)
Lindholt, et al. [39]	(+)	(−)	(−)	(−)	(−)	(+)	(−)	(+)
Memon, et al. [40]	(+)	(−)	(−)	(−)	(−)	(+)	(−)	(+)
Eilenberg, et al. [41]	(+)	(−)	(+)	(−)	(−)	(+)	(−)	(+)

Abbreviations: D1–7: Domain 1–7, (−): Low risk of bias, (+): Moderate risk of bias, (x): Serious risk of bias.
